# Dual tolerance to soil drought and excess moisture stresses in cowpea genetic resources assessed using multiple indicators

**DOI:** 10.3389/fpls.2025.1573313

**Published:** 2025-06-12

**Authors:** Kohtaro Iseki, Olajumoke Olaleye

**Affiliations:** ^1^ Japan International Research Center for Agricultural Sciences (JIRCAS), Tsukuba, Ibaraki, Japan; ^2^ International Institute of Tropical Agriculture (IITA), Ibadan, Oyo, Nigeria

**Keywords:** drought, excess moisture, dual-tolerance, cowpea, wild species, root morphology

## Abstract

**Introduction:**

Climate change poses significant challenges to agriculture, particularly for upland crops in vulnerable regions. Cowpeas (*Vigna unguiculata*), a vital protein source in the dry savannah of West Africa, face production constraints due to yield variability from inconsistent rainfall patterns. Projections indicate an increase in extreme rainfall events, exacerbating excess moisture stress and complicating cultivation. This study evaluated the dual tolerance of cowpeas to both drought and excessive moisture by examining 99 genetic accessions, including both cultivated varieties and wild ancestors.

**Methods:**

A total of 99 cowpea accessions, comprising 54 cultivated accessions (*Vigna unguiculata* [L.] Walp.) and 45 wild ancestor accessions, were analyzed. Combinations of multiple indices with large genotypic variation—such as chlorophyll fluorescence, SPAD readings, and shoot biomass—were used to assess stress tolerance.

**Results:**

While most accessions showed tolerance to only one stress or neither, ten accessions exhibited dual tolerance. Of the ten, nine were wild ancestors, underscoring the potential of wild genetic resources for crop improvement. As a factor underlying dual tolerance, we focused on the root morphological plasticity, allowing dynamic structural adjustments to different soil water conditions. Under excess moisture, a dual-tolerant accession formed lysigenous aerenchyma, facilitating oxygen diffusion and nitrogen fixation, while under drought conditions, it increased stele proportion. In contrast, a cultivar sensitive to both stressors exhibited lower plasticity, limiting its adaptability.

**Conclusion:**

This study highlights the importance of using multiple indices to assess stress tolerance, as different parameters reflect distinct physiological responses. The findings provide valuable insights for breeding climate-resilient cowpea varieties that can adapt to fluctuating soil water conditions.

## 1 Introduction

Cowpea (*Vigna unguiculata* (L.) Walp) is one of the world’s major dry beans, cultivated across diverse climate zones from arid to humid regions. Due to its adaptability to dry conditions, cowpea serves as a crucial energy source, particularly in the dry savannas of West Africa, where it contributes 16% of the protein intake (FAOSTAT; https://www.fao.org/faostat/en/#data). Over the past 55 years, cowpea grain yield in West Africa has more than doubled; however, the 10-year average yield (0.51 t ha^-1^) remains significantly below the crop’s potential yield (2–3 t ha^-1^). In this region, cowpea cultivation is highly dependent on natural rainfall, and unstable rainfall patterns result in annual yield variations of 11–23%, considerably higher than the 6–10% reported in North America (FAOSTAT).

Future rainfall patterns in the Sudan Savanna region of West Africa present a complex scenario. Earlier predictions indicated a potential decline in rainfall, leading to an estimated 18% reduction in crop production (Roudier et al., 2011). However, recent projections using the CMIP6 suggest an increase in total rainfall during the cowpea growing season, with a higher frequency of heavy rainfall events expected over the next 30 years. This could paradoxically result in yield reductions due to excess moisture stress (Iizumi et al., 2024). Field studies conducted in the central Sudan Savanna region support these projections, showing that even temporary occurrences of excess soil moisture negatively impact cowpea growth (Iseki et al., 2021) and reduce fertilizer effectiveness (Iseki et al., 2023). Given the uncertainties in long-term climate predictions, agricultural strategies should work to address the dual challenges of drought and excessive moisture stresses. Although drought tolerance has long been a major focus of regional cowpea breeding programs (Boukar et al., 2019), tolerance to excess moisture stress has received little attention. Integrating drought and excess moisture tolerance into breeding programs is increasingly critical to enhance the resilience of cowpea to the unpredictable and extreme rainfall conditions associated with climate change.

Several mechanisms are shared between excess moisture and drought tolerance. One example is the formation of root cortical aerenchyma, which reduces the metabolic cost of sustaining root tissues and promotes root growth under water-limiting conditions (Vadez, 2014). Maize genotypes with high aerenchyma formation have shown up to an eight-fold increase in yield under drought stress compared to those with low aerenchyma formation (Lynch et al., 2014). Similarly, Ye et al. (2018) identified a quantitative trait locus in soybean that increased adventitious root formation for waterlogging tolerance and conferred drought tolerance. These findings suggest that dual tolerance to both stresses may be achievable. However, studies have also reported trade-offs between drought and excess moisture tolerance in 806 tree and shrub species (Niinemets and Valladares, 2006) and in elite maize inbred lines (Zaidi et al., 2008). The ecological and physiological mechanisms underlying dual tolerance remain unclear, emphasizing the need for systematic studies using diverse genetic resources to better understand and address both types of stress tolerance.

A major constraint in evaluating genetic resources for dual tolerance is the lack of standardized evaluation methods. In field studies, primary indices such as grain yield are commonly used; however, these indices reflect the cumulative effects of diverse environmental factors experienced throughout the growing period. Consequently, they are not well suited for precise phenotypic evaluation of the specific effects of experimental soil water treatments. In contrast, secondary indices—such as chlorophyll fluorescence and chlorophyll content (e.g., SPAD values)—reflect the plant physiological status at the moment of stress exposure (Souza et al., 2004). Although these indices may not directly correlate with the final yield, they are highly sensitive and repeatable, making them useful tools for physiologically based phenotyping under controlled experimental conditions.

However, secondary indices often lead to discrepancies across evaluation results. For example, while both chlorophyll fluorescence and SPAD are widely used as drought tolerance indicators (Ravelombola et al., 2020; Singh and Reddy, 2011), their responses to drought stress are not always consistent. A decrease in chlorophyll fluorescence may not correspond to a decrease in SPAD values under varying drought intensities (Arief et al., 2023). Moreover, the appropriate index for tolerance evaluation should be specific to the stress being assessed (Su et al., 2015). Therefore, selecting suitable indices that align with prevailing stress conditions is crucial to ensure accurate and reproducible evaluations.

In this study, cowpea genetic resources, including the wild ancestor *Vigna unguiculata* subsp. *dekindtiana*, were evaluated for tolerance to excess moisture and drought. The subspecies *dekindtiana* is recognized as a likely progenitor of domesticated cowpeas (Huynh et al., 2013). These wild species, which inhabit geographically and climatically diverse regions, may offer valuable genetic traits for broader adaptation to environmental stresses compared to cultivated varieties (Tomooka et al., 2011). To address inconsistencies across indices, this study employed multiple indices tailored to excess moisture and drought treatments. The objective was to clarify the presence of dual tolerance and to characterize the root morphological adaptations associated with these stresses.

## 2 Materials and methods

### 2.1 Plant materials and growth conditions

This study used a total of 99 cowpea accessions, consisting of 54 cultivated accessions (*Vigna unguiculata* [L.] Walp.) and 45 wild ancestor accessions (*Vigna unguiculata* subsp. *dekindtiana*) (**Supplementary Table S1**). The cultivated accessions were selected from the cowpea mini-core collection developed by the International Institute of Tropical Agriculture (IITA) (Fatokun et al., 2018), while the wild ancestor accessions were obtained from the IITA Genetic Resources Center. These wild accessions represent a range of geographical origins with varying climate conditions, from dry to wet regions. Both cultivated and wild accessions were single-seed descent lines with genetically fixed genomic regions to minimize phenotypic segregation.

Pot experiments were conducted twice in a glasshouse at IITA in Ibadan, Nigeria (7°29′ N, 3°54′ E). The plants were grown in long plastic pots (9 cm in diameter, 20 cm in height) filled with sterilized topsoil (sandy loam soil, pH of 7.6) containing 2.0 g/kg organic carbon, 0.40 g/kg nitrogen, and 3.8 mg/kg Bray-1 phosphate. Seeds were sown on October 12, 2018, and January 18, 2019, at a rate of six seeds per pot. After full emergence, the plants were thinned to three per pot. Nine pots were prepared for each accession in each experiment, resulting in a total of 891 pots.

The pots were placed in wooden boxes (6 m long, 25 cm wide, and 25 cm high), lined with a waterproof sheet, and filled with 2 cm of water to ensure uniform irrigation until water treatments began (**Supplementary Figure S1**). Two boxes were used for each treatment and replicate. Water treatments were initiated two weeks after sowing, when the first trifoliate leaves were fully expanded. Three pots per accession were assigned to each treatment: excess moisture, drought, and control. For the excess moisture treatment, the water depth in the box was increased to 18 cm. For the drought treatment, all water was drained from the box. The control pots were maintained under the same conditions as before the treatments. The experimental design followed a randomized block arrangement with three replicates.

**Figure 1:**
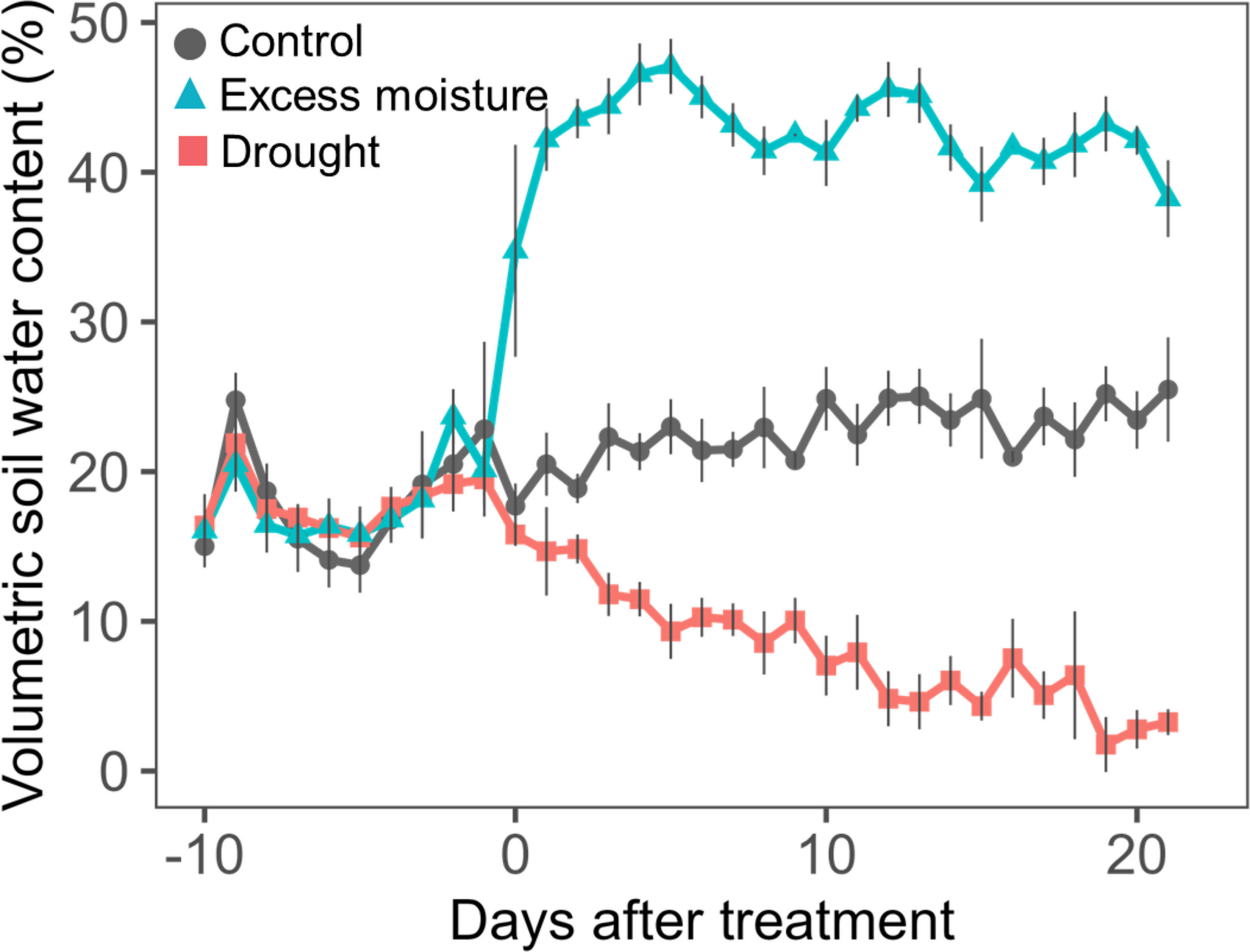
Temporal changes in volumetric soil water content for control, excess moisture and drought treatments during experimental periods. Values are the mean and standard error of three replicates and two experimental replicates (n = 6).

Volumetric soil water content was monitored in three unplanted pots placed in each wooden box. Measurements were taken every 2–3 days using a soil moisture sensor (ECH2O EC–5, METER, Pullman, WA, USA). During the treatments, the average soil water contents were 23.1% under control conditions, 42.3% under excess moisture, and 6.9% under drought (**Figure 1**). The average minimum and maximum temperatures recorded were 31.5 ± 1.5/22.5 ± 3.4°C for the first experiment and 35.2 ± 2.1/23.6 ± 1.6°C for the second experiment.

### 2.2 Plant evaluation

The plant physiological status concerning photosynthesis was evaluated using parameters of chlorophyll fluorescence and chlorophyll content. Chlorophyll fluorescence measurements were taken three times over a 2–3-week period following the initiation of water treatments, using a FluorPen FP100 (Photon Systems Instruments, Drásov, Czech Republic). The maximum quantum yield of photosystem II under light-adapted conditions (Fv’/Fm’) was used to evaluate leaf physiological status in relation to photosynthetic electron transport. Measurements were conducted between 10:00 and 14:00. Chlorophyll content was measured on the same days as the fluorescence measurements using a SPAD-502 Plus meter (KONICA MINOLTA Inc., Tokyo, Japan). Measurements were performed on fully opened, light-adapted uppermost leaves from three different plants per pot. Four weeks after initiation of water treatments, the shoots of all three plants in each pot were harvested. The harvested plant material was dried at 70°C for more than 48 h and then weighed on an electronic scale (TX2202N; SHIMADZU, Kyoto, Japan) to determine shoot biomass.

### 2.3 Definition of tolerance to excess moisture and drought stress

The values for chlorophyll fluorescence, SPAD readings, and shoot biomass were averaged across measurements, plant replicates, and the two experimental replicates. Relative values for each accession and treatment were calculated using the control as a baseline. Among these three indices, those showing large genotypic variation in relative values were selected for further analysis: shoot biomass and SPAD for excess moisture tolerance, and chlorophyll fluorescence and SPAD for drought tolerance.

Accessions were classified into three categories based on their relative values—tolerant, less tolerant, and sensitive. Tolerant accessions were defined as those with both relative values higher than the average. Less tolerant accessions had one of the two indices above the average. Sensitive accessions had both indices below the average. This classification was applied separately for each water treatment. Based on their tolerance to excess moisture and drought, the 99 accessions were further divided into five groups using K-means clustering, performed using the statistical software R (version 4.2.1).

### 2.4 Root anatomical analysis

Two accessions, one tolerant (TVNu362, wild ancestor) and one sensitive (TVu3745 cultivar) to both excess moisture and drought, were selected based on clustering results. These plants were grown under the same experimental conditions as described for the tolerance evaluation, using long plastic pots and wooden boxes. Three seeds were sown per pot on January 10, 2022, and thinned to one plant per pot after full emergence. A total of 45 pots were prepared for each accession, with five pots per treatment (control, excess moisture, and drought) and three replicates. Excess moisture and drought treatments began two weeks after sowing. The average soil water content during the treatments was 20.4% for the control, 37.1% for excess moisture, and 2.9% for drought conditions.

Two weeks after the water treatments, the soil and roots were carefully removed from the pots. The soil was gently washed away to collect intact roots. A 1-cm segment of the taproot at 4–5 cm below the soil surface was sampled according to the previous study (Thomas et al., 2005). The segments were then submerged in phosphate buffer (pH 7.2) containing 4% glutaraldehyde for fixation. The samples were vacuum-infiltrated for two hours to ensure complete tissue fixation. Dehydration was performed through sequential immersion in 20%, 40%, 60%, and 80% ethanol solutions, each for 12 h. Cross-sections were prepared using a hand microtome (THK, Kenis, Osaka, Japan) and sectioned with a razor blade. Sections were imaged with an optical microscope (AS-Z40-L; MicroAdvance, Osaka, Japan) equipped with a CCD camera (AS-300C-D; MicroAdvance). Eight sections were taken from different parts of each sample and photographed. In total, 720 sectional images were obtained, representing two accessions, three treatments, five plants, three replicates, and eight sections per sample. Root radius, stele radius, and the presence of cortical aerenchyma were measured for each image using the image analysis software ImageJ (version 1.43u; National Institutes of Health). The cortex/stele ratio was calculated as (root radius – stele radius)/stele radius.

### 2.5 Statistical analysis

Statistical analyses were conducted using R (version 4.2.1). The effects of cultivation replicates, accession, and water treatment on the indices were evaluated using a multiway analysis of variance (ANOVA). The F-values and percentage of contribution (ρ) were calculated from type-II sum of squares and mean squares and then compared among the factors. Tukey’s multiple comparison tests were performed to determine significant differences in chlorophyll fluorescence, SPAD readings, and shoot biomass among the treatments and accessions. Welch’s t-test was used to determine significant differences between cultivated and wild ancestor accessions. Additionally, multiple comparison tests were applied to evaluate significant differences in root radius, stele radius, cortex/stele ratio, and aerenchyma emergence ratio among different treatments and accessions. The aerenchyma emergence ratio represents the percentage of sections showing aerenchyma formation out of a total of 40 sections (derived from five plants with eight sections each), assessed across three replications.

## 3 Results

### 3.1 Plant responses to drought and excess moisture

The values of quantum yield, SPAD readings, and shoot biomass showed minimal variation, attributable to cultivation replicates, compared to the effects of accession and water treatments (**Supplementary Table S2**). Therefore, data from different cultivation replicates were averaged for subsequent analyses. The mean values for quantum yield, SPAD readings, and shoot biomass of both cultivars and wild ancestors are summarized in **Table 1**; **Supplementary Table S1**. Under control conditions, wild ancestors exhibited higher quantum yield, lower SPAD values, and smaller shoot biomass compared to the cultivars. To account for differences in baseline values across accessions, relative values of excess moisture to control and drought to control were calculated for each accession. Plant damage from water treatments was assessed based on the reduction in these relative values, which would remain close to one if plants were unaffected by the treatment. Since no correlations were observed within these three traits (**Supplementary Figure S2**), each trait can be considered as an independent index with different meanings.

**Table 1:** Indices used for tolerance evaluation under drought and excess soil moisture conditions.

Soil water conditions	Fv’/Fm’			SPAD			Shoot dry weight (g plant^-1^)		
	Cultivar	Wild ancestor	Significant difference between the species	Cultivar	Wild ancestor	Significant difference between the species	Cultivar	Wild ancestor	Significant difference between the species
Control	0.665 ± 0.036 ^a^	0.703 ± 0.063 ^a^	**	42.7 ± 10.4 ^a^	33.7 ± 6.9 ^a^	**	3.68 ± 1.66 ^a^	1.35 ± 1.1 ^a^	**
Excess moisture	0.667 ± 0.035 ^a^	0.713 ± 0.050 ^a^	**	36.4 ± 9.5 ^b^	29.1 ± 7.1 ^b^	**	2.52 ± 0.78 ^b^	1.17 ± 0.68 ^a^	**
Drought	0.301 ± 0.150 ^b^	0.537 ± 0.172 ^b^	**	31.5 ± 14.5 ^c^	30.1 ± 7.2 ^b^	ns	0.32 ± 0.12 ^c^	0.28 ± 0.22 ^b^	ns
Excess moisture /Control	1.01 ± 0.05 ^A^	1.02 ± 0.07 ^A^	ns	0.87 ± 0.21 ^A^	0.89 ± 0.15 ^A^	ns	0.68 ± 0.22 ^A^	0.83 ± 0.26 ^A^	**
Drought/Control	0.46 ± 0.17 ^B^	0.77 ± 0.15 ^B^	**	0.76 ± 0.27 ^B^	0.89 ± 0.18 ^A^	**	0.1 ± 0.08 ^B^	0.29 ± 0.23 ^B^	**

Values are means ± standard deviation for 54 accessions of cultivars and 44 accessions of wild ancestors.

** and ns represent that the differences between cultivar and wild ancestor were significant at the P < 0.05 level and not significant, respectively.

Different lowercase and uppercase letters represent significant differences between the soil water conditions (P < 0.05) for values and relative values, respectively.

Under excess moisture conditions, the relative quantum yield values remained largely unaffected in both cultivars and wild ancestors. Similarly, the relative SPAD values showed only moderate reductions, averaging from 0.76 to 0.89 across accessions. Shoot biomass also decreased under excess moisture, though the reduction was less pronounced compared to that under drought conditions.

**Figure 2:**
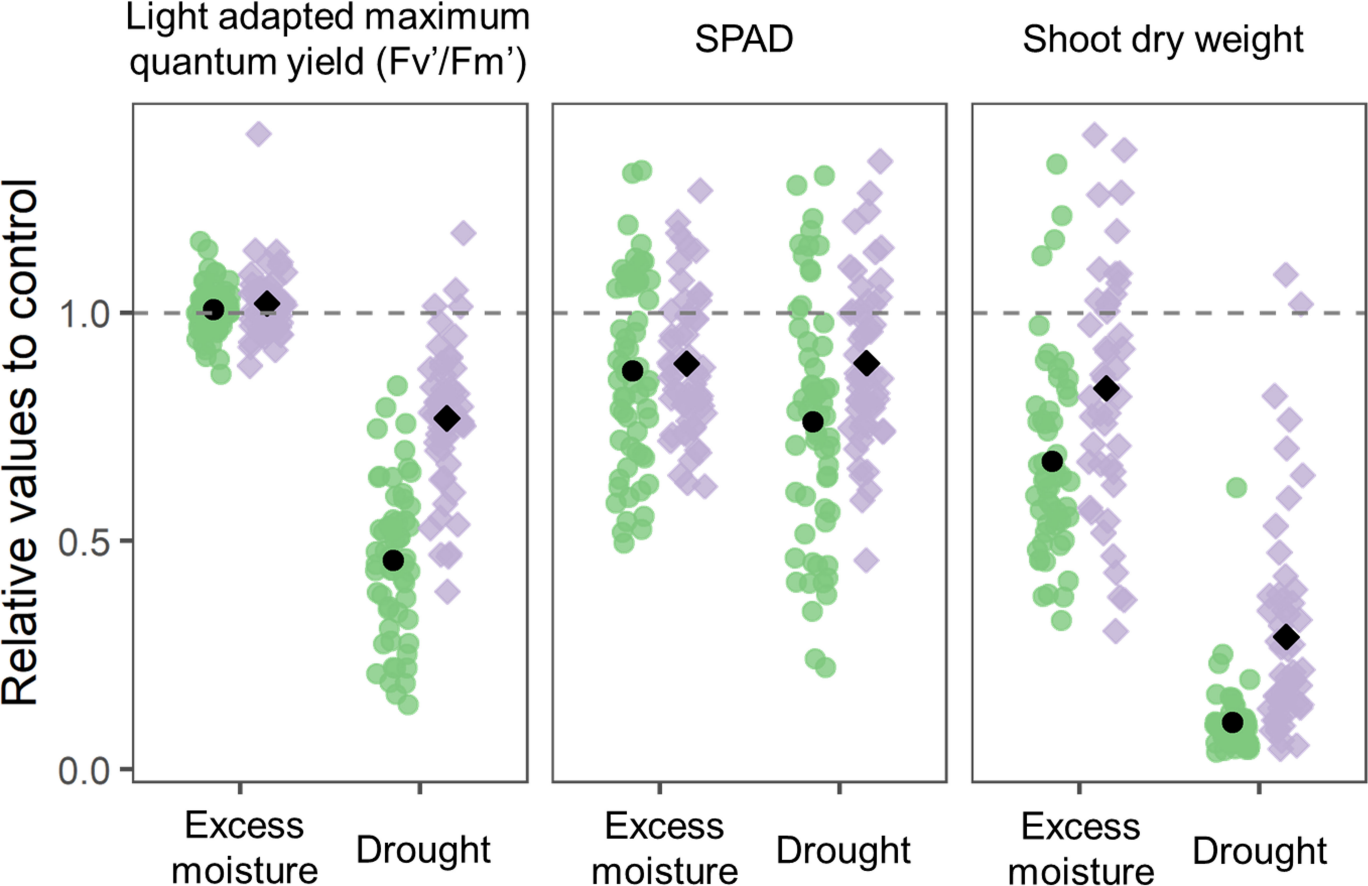
Distribution of the indices for 54 cultivars and 44 wild ancestors under excess moisture and drought conditions. Each data point represents the mean value relative to the control for three replications and two experimental replicates (n = 6). The values for cultivars and wild accessions are shown separately in green and purple, respectively. The black circles and squares represent the overall averages for cultivars and wild ancestors, respectively. The dashed horizontal line at the relative value of 1.0 indicates the average under control conditions.

In contrast, drought conditions led to a notable decline in physiological parameters. The relative quantum yield decreased to an average of 0.46 in cultivars and 0.77 in wild ancestors (**Figure 2**). SPAD values exhibited a similar declining trend and shoot biomass was substantially reduced, with a more severe impact observed in cultivars than in wild ancestors.

Based on these responses, SPAD and shoot biomass were selected as tolerance indices for excess moisture, as they reflected genotypic variations. For drought tolerance, SPAD and quantum yield were chosen due to their marked and consistent responses. Although shoot biomass was strongly affected by drought, it was excluded from further drought tolerance evaluation due to its relatively low genotypic variation compared to the other indices.

### 3.2 Detection of tolerant accessions

**Figure 3:**
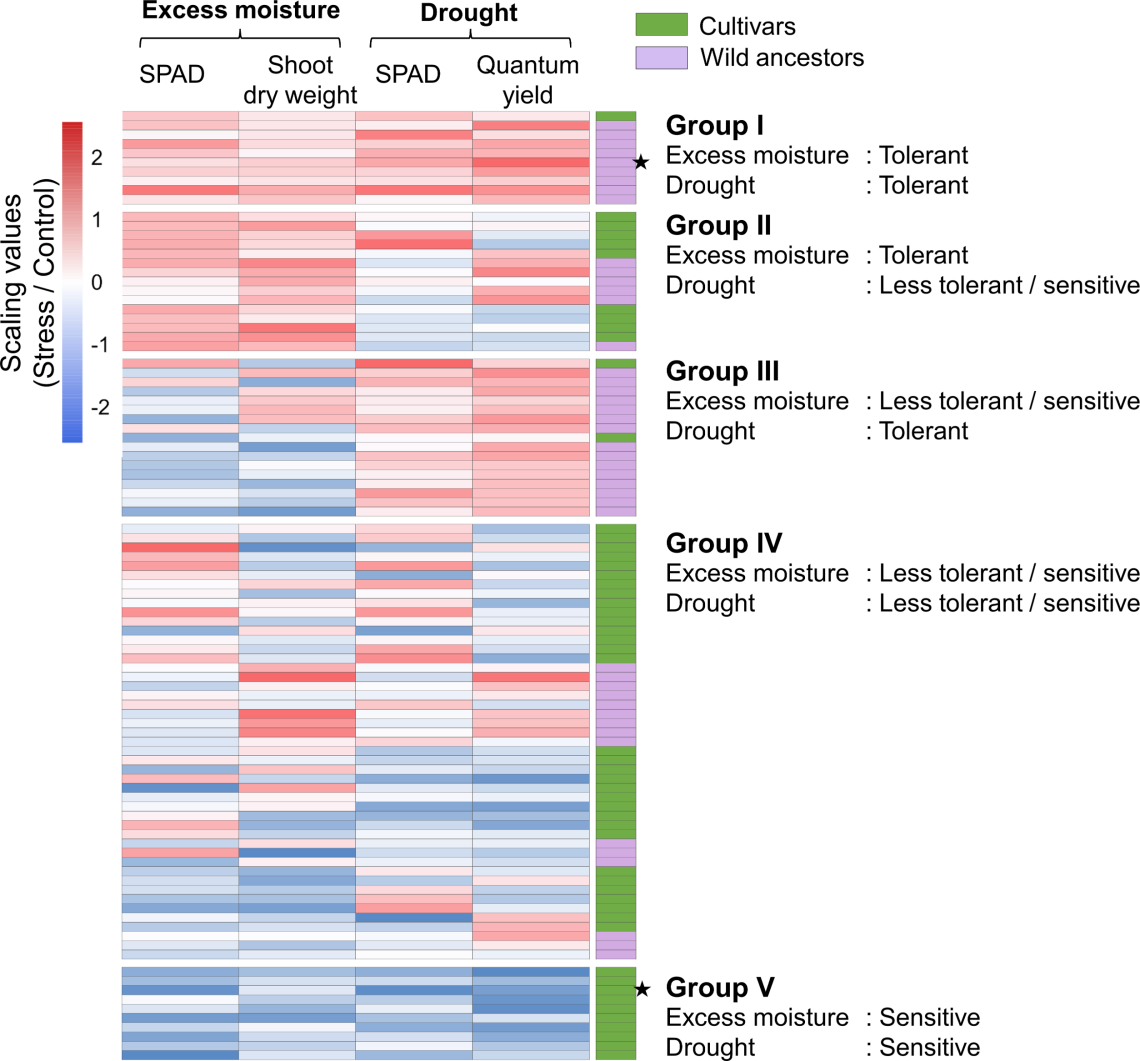
Grouping of the 99 accessions based on the tolerance to excess moisture and drought stresses. Five cluster groups with different responses to excess moisture and drought stress were identified. Deeper red and blue represent higher and lower relative values for each index, respectively. Cultivars and wild ancestors are shown in green and purple, respectively. Accessions marked with black stars were selected for root anatomical analysis.

Based on the relative trait values under each water treatment, the 99 accessions were categorized into five groups reflecting distinct tolerance profiles (**Figure 3**). Group I included accessions tolerant to both excess moisture and drought, comprising one cultivar and nine wild ancestors; Group II included accessions tolerant to excess moisture but less tolerant or sensitive to drought, comprising nine cultivars and six wild ancestors; Group III included accessions tolerant to drought but less tolerant or sensitive to excess moisture, comprising two cultivars and 15 wild ancestors; Group IV included accessions with less tolerant or sensitive to both stress conditions, comprising 32 cultivars and 15 wild ancestors; finally, Group V included the sensitive accessions to both excess moisture and drought, comprising 10 cultivars.

Under excess moisture conditions, accessions exhibiting tolerance included members of Groups I and II, while under drought conditions, tolerant accessions were primarily found in Groups I and III. Overall, drought-tolerant accessions were predominantly wild ancestors, whereas tolerance to excess moisture was observed in both cultivars and wild ancestors

### 3.3 Root anatomical changes in response to soil water conditions

**Figure 4:**
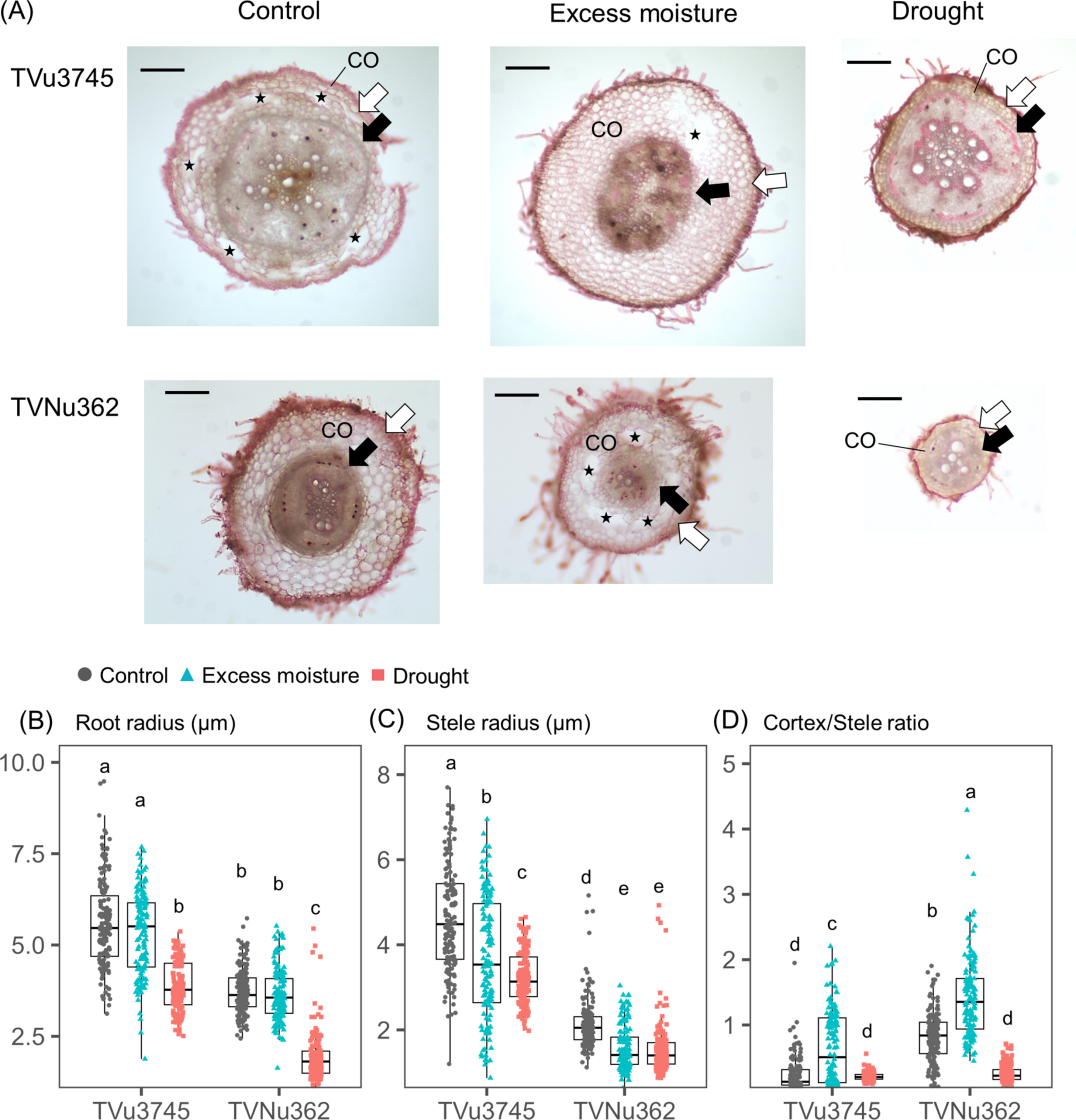
Root morphological changes of dual-tolerant (TVNu362) and dual-sensitive (TVu3745) accessions in response to excess moisture and drought conditions. **(A)** Anatomical images of root cross section. CO represents cortex layer determined as the proportion between epidermis (white arrow) and endodermis (black arrow). The black-colored star represents the cortex lysigenous aerenchyma. Bars are the reference scale of 200 μm. **(B)** Distributions in root radius, **(C)** stele radius, and **(D)** cortex/stele ratio. Gray circles, green triangles, and red squares are the points of color, excess moisture, and drought conditions, respectively. Different letters represent significant differences among the accessions and soil water conditions at the P < 0.05 level.

Accessions from Group I (TVNu362, wild ancestor) and Group V (TVu3745, cultivar) were selected for root anatomical analysis. The root and stele radius were measured under control, excess moisture, and drought conditions (**Figure 4A**). The cortex is defined as the tissue layer between the epidermis and endodermis; therefore, a higher cortex/stele radius ratio indicates a thicker cortex. The aerenchyma was only observed in the lysigenous form within the cortex. The root radius was consistently larger in TVu3745 than in TVNu362 across all water treatments. Its distribution remained similar between control and excess moisture conditions but decreased significantly under drought (**Figure 4B**). The average reduction in root radius was greater in TVNu362 (-48%) than in TVu3745 (-31%). Similarly, the stele radius was larger in TVu3475 than in TVNu362. It decreased under both excess moisture and drought conditions with minimal differences between these treatments (**Figure 4C**). The cortex/stele ratio was higher in TVNu362 than in TVu3745 under control conditions (**Figure 4D**) and increased significantly in both accessions under excess moisture conditions. Under drought, this ratio declined by 70% in TVNu362 but showed no significant change in TVu3745.

**Figure 5:**
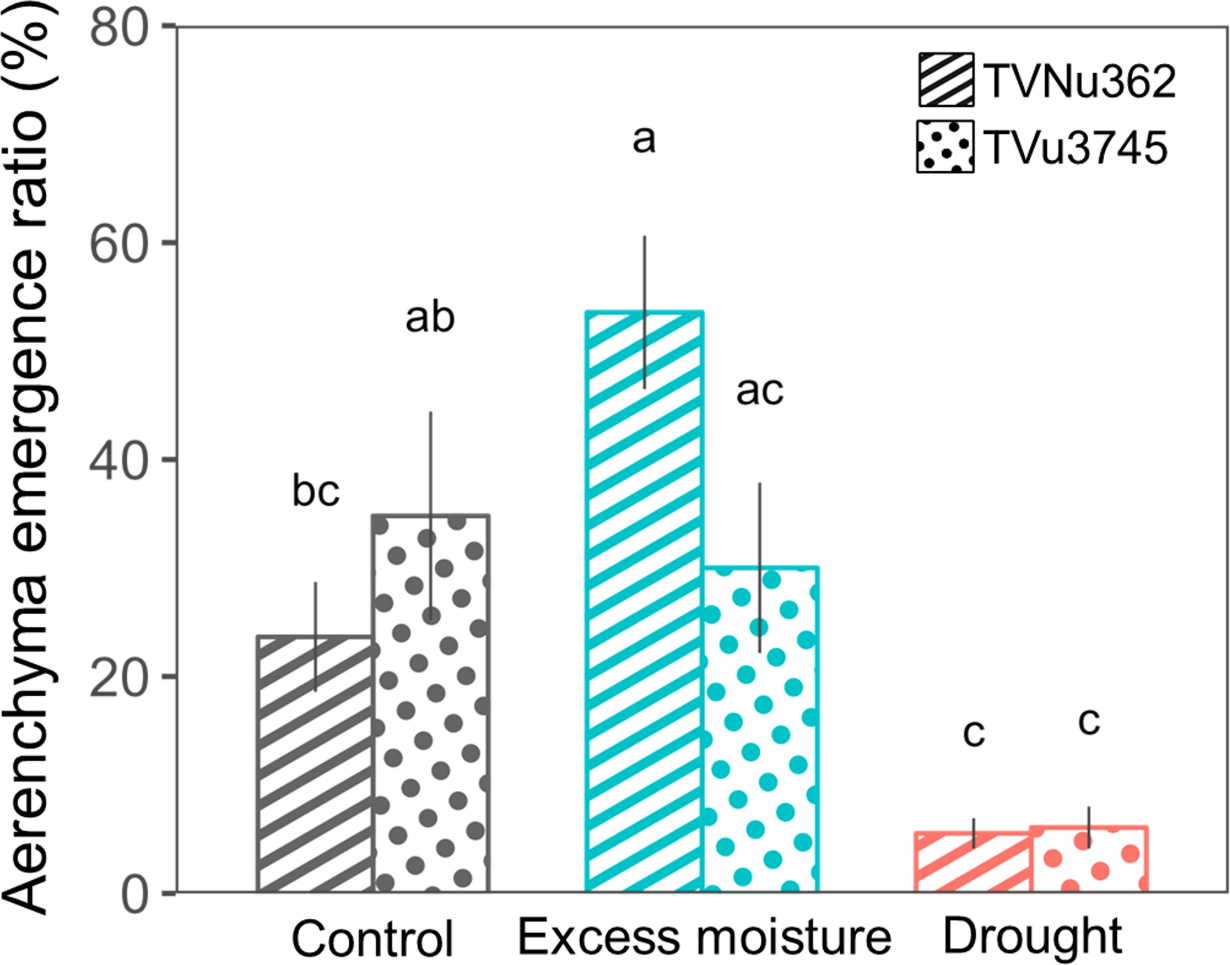
Changes in the emergence ratio of cortical lysigenous aerenchyma in a dual-tolerant (TVNu362) and dual-sensitive (TVu3745) accession in response to excess moisture and drought conditions. Means and standard errors of five replicates of the aerenchyma emergence ratio are shown. The ratio of each replicate was obtained from 40 samples consisting of 8 sections and 5 plants. Different letters represent significant differences between the soil water treatments and accessions (P < 0.05).

The emergence ratio of root cortical aerenchyma is summarized in **Figure 5**. In TVu3745, the emergence ratio was similar for the control (34.8%) and excess moisture (30.0%) treatments. In contrast, the ratio in TVNu362 more than doubled, increasing from 23.6% under control conditions to 53.6% under excess moisture. Under drought, the emergence ratio dropped significantly to 5.5% in TVNu362 and 6.1% in TVu3745.

## 4 Discussion

Among the ten accessions in Group I that demonstrated dual tolerance to both stresses, nine were wild cowpea ancestors, highlighting the potential of wild genetic resources for crop improvement under increasingly variable soil water conditions driven by climate change.

While most drought-tolerant accessions (Group III) were wild ancestors, half of the accessions tolerant only to excess moisture stress (Group II) were cultivated varieties. This suggests that the genetic diversity loss during domestication, commonly referred to as the “domestication bottleneck” (Dempewolf et al., 2017), had less impact on traits associated with excess moisture tolerance than on those related to drought tolerance.

One possible factor contributing to the dual tolerance observed in Group I is high root morphological plasticity (**Figure 4**). This trait likely enables plants to dynamically adjust their root architecture in response to varying soil water conditions, thereby optimizing oxygen supply under excess moisture and enhancing water uptake during drought stress (Yamauchi et al., 2021a). The importance of root anatomical plasticity under contrasting moisture conditions has been reported in Phragmites species, where adaptation to low-oxygen environments involves efficient modulation of the cortex-to-stele ratio (Yamauchi et al., 2024). These findings suggest that root morphological plasticity may represent a broadly applicable strategy across both Poaceae and Fabaceae. However, it should be emphasized that other physiological or morphological mechanisms, including the adaptation of above-ground parts, may also contribute to the observed dual tolerance.

Under excess moisture conditions, TVNu362, a dual-tolerant accession (wild ancestor), increased its cortical proportion and developed well-formed lysigenous aerenchyma (**Figures 4**, **5**), likely maintaining oxygen diffusion and supporting nodule activity for nitrogen fixation (Thomas et al., 2005; Shimamura et al., 2002). Unlike soybeans, which form secondary aerenchyma after prolonged flooding (Yamauchi et al., 2013), cowpeas primarily rely on lysigenous aerenchyma formation as a tolerance mechanism to excess moisture. In contrast, TVu3745, a dual-sensitive accession (cultivar) also exhibited cortical expansion under excess moisture (**Figure 4**) but failed to develop sufficient aerenchyma (**Figure 5**). This suggests that despite structural changes, the roots of the dual-sensitive cultivar could not efficiently supply oxygen, which may have limited its tolerance to excess moisture.

Under drought conditions, the dual-tolerant accession exhibited an increase in stele proportion (**Figure 4**), thereby enhancing water transport efficiency relative to the cost of root production. This structural adjustment facilitates sustained water uptake under low soil moisture conditions (Klein et al., 2020). In contrast, the dual-sensitive accession responded by producing thinner roots without a significant change in stele proportion. Previous studies have suggested that the cortex-to-stele ratio, rather than the overall root cross-sectional area, plays a key role in adaptation to varying soil water conditions (Yamauchi et al., 2021b). The lack of changes in the stele proportion may negatively affect their adaptation to the drought conditions.

Under control conditions, the dual-tolerant accession exhibited a root structure more adapted to excess moisture, characterized by a higher cortical proportion (**Figure 4**). In contrast, the dual-sensitive cultivar had a root structure better suited to drought, with a higher stele proportion. Interestingly, the dual-sensitive cultivar displayed a higher frequency of aerenchyma formation even under control conditions (**Figure 5**). This could represent an adaptation to reduce cortical tissue costs in dry environments, as suggested by Vadez (2014). These findings suggest that although the dual-sensitive accession may be inherently adapted to moderate drought conditions, this pre-existing trait limits the plant’s ability to develop additional aerenchyma in response to excess moisture. Furthermore, the inherently lower cortical proportion of the dual-sensitive cultivar under the control conditions leaves minimal room for structural modification under varying moisture conditions. In contrast, the dual-tolerant accession, which naturally has a thicker cortex, shows greater flexibility. It can form lysigenous aerenchyma under excess moisture and reduce its cortical proportion under drought conditions.

This study employed two distinct indices, both showing significant variation among accessions, to evaluate tolerance. This approach provides a more reliable assessment of genetic resources than single-index evaluations. Striker and Colmer (2017) evaluated excess moisture tolerance in 17 legume species using relative shoot biomass and reported a wide range of tolerance, from 40% to 101%. In this study, the average relative shoot biomass of the wild ancestors was 87%, compared to 68% in the cultivars (**Table 1**). These results indicate that the cowpea accessions examined in this study demonstrated relatively high tolerance to excess moisture compared to other legumes. However, some accessions exhibited high relative shoot biomass but low SPAD values under excess moisture conditions (**Figure 3**), suggesting that although shoot growth was maintained, physiological activity was impaired. These findings emphasize that ranking tolerance based on a single index may be insufficient, highlighting the importance of integrating multiple indicators for a more comprehensive evaluation.

The contrasting responses of SPAD values and shoot biomass observed in Groups III and IV under excess moisture stress indicate variations in stress response mechanisms among accessions (**Figure 3**). Some genotypes showed a greater decline in SPAD values, while others showed a larger reduction in shoot biomass. SPAD values are generally associated with chlorophyll content and nitrogen concentration (Xiong et al., 2015), both of which can be affected by excess moisture stress through inhibition of nitrogen fixation (Maekawa et al., 2011). These patterns suggest differing strategies in maintaining growth or preserving leaf nitrogen status under stress. Under drought conditions, chlorophyll fluorescence typically declines earlier than SPAD, indicating an early reduction in photosynthetic efficiency (Juzoń et al., 2019). However, some genotypes exhibited a reverse pattern. The suppression of light energy absorption as a mechanism to avoid oxidative damage may account for such responses (Lima et al., 2018). These results highlight the need to evaluate multiple physiological indices to capture the diverse stress adaptation strategies among genotypes.

## 5 Conclusions

While cowpea is typically cultivated in dryland regions, this study demonstrates its diverse tolerance not only to drought but also to excess moisture. Dual-tolerant cowpea accessions, which dynamically adjust their root structure, represent valuable genetic resources for breeding climate-resilient varieties. While various mechanisms may underlie their adaptability to different soil water levels, our findings highlight a possible role of root morphological plasticity in developing dual tolerance to both drought and excess moisture. Balancing oxygen supply under excess moisture and water uptake during drought is critical for adapting to the increasingly unpredictable rainfall patterns caused by climate change. However, there may be negative aspect associated with this adaptation. Roots with a high cortical proportion may have reduced nutrient uptake and elongation capacity under normal conditions, suggesting that dual-tolerant accessions might compensate for this through improved above-ground nutrient and water use efficiency. This warrants further investigation. Evaluating stress tolerance using multiple indices, as demonstrated in this study, provides a more reliable assessment of genetic resources by capturing diverse tolerance mechanisms.

## Data Availability

The raw data are available in **Supplementary Table S1** without undue reservation.
