# Supplementary figures and images for: Dual tolerance to soil drought and excess moisture stresses in cowpea genetic resources assessed using multiple indicators
Source: Front Plant Sci. 2025 Jun 12;16:1573313. doi: 10.3389/fpls.2025.1573313 (PMC12198204; doi:10.3389/fpls.2025.1573313)

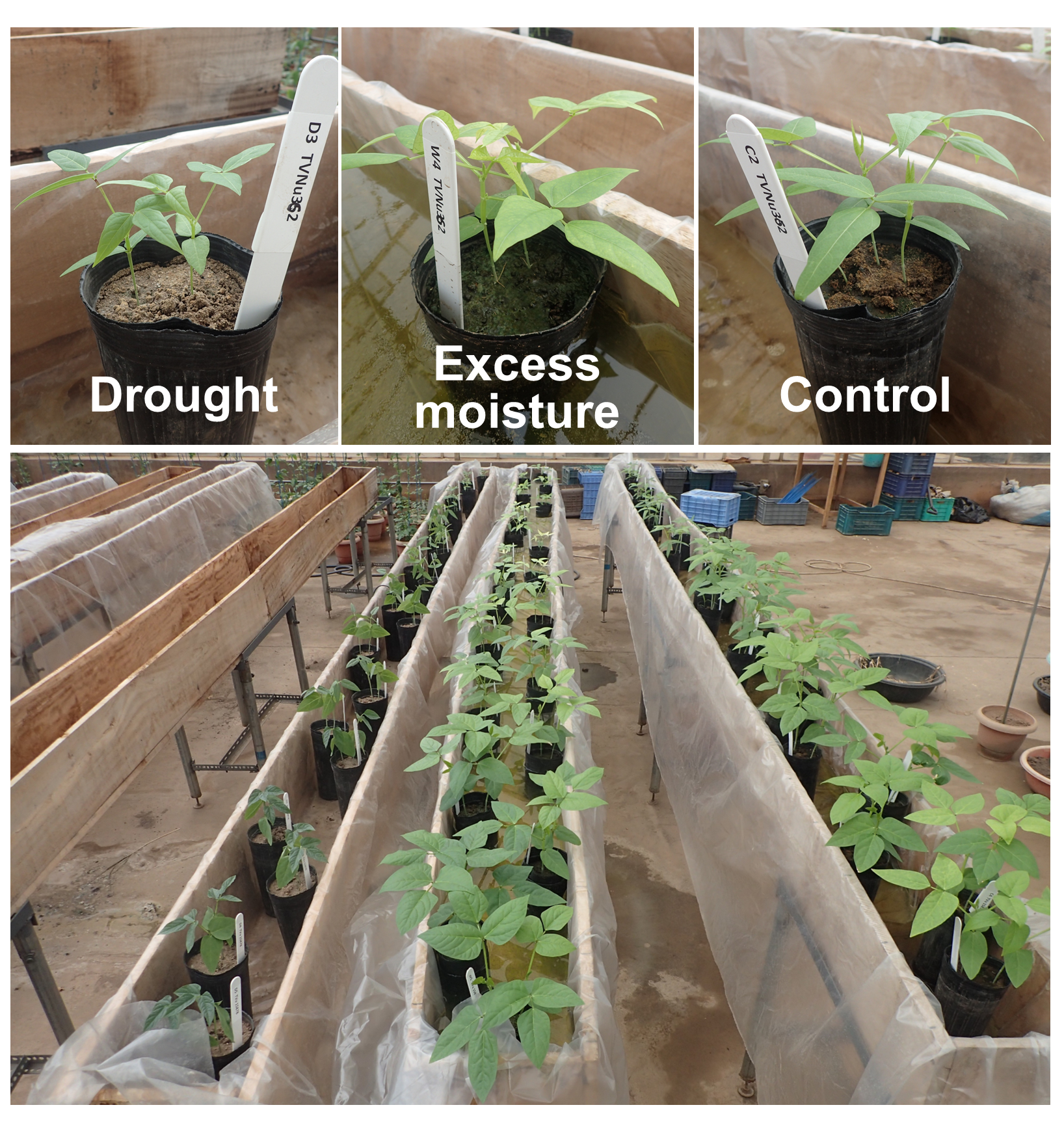


Supplemental Figure S1. Condition of the plants during cultivation.

Supplement: Supplementary file 1 [file DataSheet1.docx]
