# Supplementary material for: Dual tolerance to soil drought and excess moisture stresses in cowpea genetic resources assessed using multiple indicators
Source: Front Plant Sci. 2025 Jun 12;16:1573313. doi: 10.3389/fpls.2025.1573313 (PMC12198204; doi:10.3389/fpls.2025.1573313)
Supplement: Supplementary file 2 [file DataSheet2.docx]

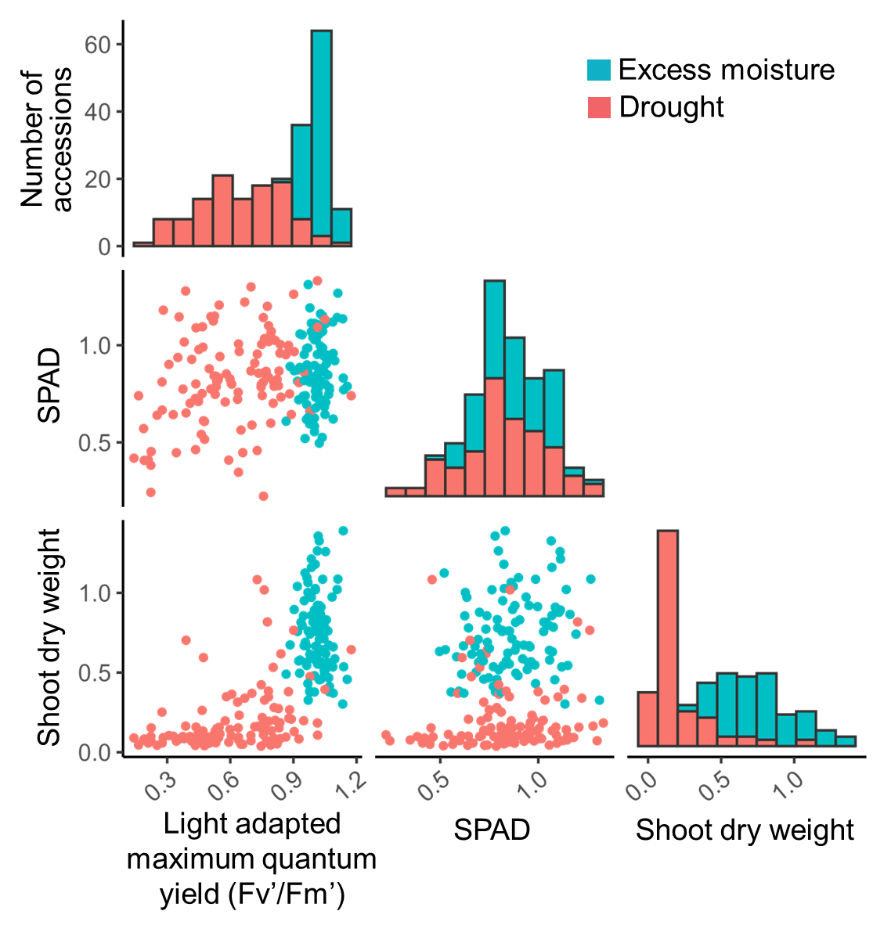


**Supplemental Figure S2. Correlation matrix among the values of chlorophyll fluorescence, SPAD, and shoot dry weight.** The distribution and correlation of 99 accessions were shown separately for excess moisture conditions and drought conditions.
