# Supplementary material for: Dual tolerance to soil drought and excess moisture stresses in cowpea genetic resources assessed using multiple indicators
Source: Front Plant Sci. 2025 Jun 12;16:1573313. doi: 10.3389/fpls.2025.1573313 (PMC12198204; doi:10.3389/fpls.2025.1573313)
Supplement: Supplementary file 4 [file Table2.docx]

|  | Fv'/Fm' | | SPAD | | Shoot dry weight | |
| --- | --- | --- | --- | --- | --- | --- |
|  | F-value | ρ (%) | F-value | ρ (%) | F-value | ρ (%) |
| Experiment (E) | 4.0 ^*^ | 0.2 | 56.3 ^**^ | 2.8 | 10.5 ^**^ | 0.4 |
| Accession (A) | 2.2 ^**^ | 7.4 | 4.6 ^**^ | 21.4 | 4.3 ^**^ | 17.7 |
| Water treatment (W) | 459.3 ^**^ | 47.4 | 77.7 ^**^ | 7.9 | 380.9 ^**^ | 35.1 |
| E×A | 0.6 ^ns^ | 0.0 | 2.3 ^**^ | 6.9 | 0.9 ^ns^ | 0.0 |
| E×W | 1.5 ^ns^ | 0.0 | 21.0 ^**^ | 2.0 | 8.9 ^**^ | 0.7 |
| A×W | 1.3 ^*^ | 3.9 | 1.9 ^**^ | 11.2 | 1.7 ^**^ | 7.1 |
| E×A×W | 0.6 ^ns^ | 0.0 | 1.2 ^ns^ | 1.7 | 0.8 ^ns^ | 0.0 |

**Table S2. F-values and contribution (ρ) of the factors obtained via the ANOVA for the trait variations.**

**: P < 0.01, *: P < 0.05, and ns: not significant.
